# Characterization and attribution of vegetation dynamics in the ecologically fragile South China Karst: Evidence from three decadal Landsat observations

**DOI:** 10.3389/fpls.2022.1043389

**Published:** 2022-10-27

**Authors:** Jie Pei, Li Wang, Huabing Huang, Lei Wang, Wang Li, Xiaoyue Wang, Hui Yang, Jianhua Cao, Huajun Fang, Zheng Niu

**Affiliations:** ^1^School of Geospatial Engineering and Science, Sun Yat-sen University, Zhuhai, China; ^2^Key Laboratory of Natural Resources Monitoring in Tropical and Subtropical Area of South China, Ministry of Natural Resources, Zhuhai, China; ^3^State Key Laboratory of Remote Sensing Science, Aerospace Information Research Institute, Chinese Academy of Sciences, Beijing, China; ^4^International Research Center of Big Data for Sustainable Development Goals, Beijing, China; ^5^The Key Laboratory of Land Surface Pattern and Simulation, Institute of Geographical Sciences and Natural Resources Research, Chinese Academy of Sciences, Beijing, China; ^6^Institute of Karst Geology, Chinese Academy of Geological Sciences (CAGS), Karst Dynamics Laboratory, Ministry of Natural Resources (MNR) & Guangxi, Guilin, China; ^7^International Research Centre on Karst, Under the Auspices of United Nations Educational, Scientific and Cultural Organization (UNESCO), Guilin, China; ^8^Key Laboratory of Ecosystem Network Observation and Modeling, Institute of Geographical Sciences and Natural Resources Research, Chinese Academy of Sciences, Beijing, China; ^9^The Zhongke-Ji’an Institute for Eco-Environmental Sciences, Ji’an, China

**Keywords:** vegetation greenness, spatial-temporal evolution, afforestation, climate change, ecological fragile areas

## Abstract

Plant growth and its changes over space and time are effective indicators for signifying ecosystem health. However, large uncertainties remain in characterizing and attributing vegetation changes in the ecologically fragile South China Karst region, since most existing studies were conducted at a coarse spatial resolution or covered limited time spans. Considering the highly fragmented landscapes in the region, this hinders their capability in detecting fine information of vegetation dynamics taking place at local scales and comprehending the influence of climate change usually over relatively long temporal ranges. Here, we explored the spatiotemporal variations in vegetation greenness for the entire South China Karst region (1.9 million km^2^) at a resolution of 30m for the notably increased time span (1987-2018) using three decadal Landsat images and the cloud-based Google Earth Engine. Moreover, we spatially attributed the vegetation changes and quantified the relative contribution of driving factors. Our results revealed a widespread vegetation recovery in the South China Karst (74.80%) during the past three decades. Notably, the area of vegetation recovery tripled following the implementation of ecological engineering compared with the reference period (1987-1999). Meanwhile, the vegetation restoration trend was strongly sustainable beyond 2018 as demonstrated by the Hurst exponent. Furthermore, climate change contributed only one-fifth to vegetation restoration, whereas major vegetation recovery was highly attributable to afforestation projects, implying that anthropogenic influences accelerated vegetation greenness gains in karst areas since the start of the new millennium during which ecological engineering was continually established. Our study provides additional insights into ecological restoration and conservation in the highly heterogeneous karst landscapes and other similar ecologically fragile areas worldwide.

## 1 Introduction

Vegetation, an integral part of terrestrial ecosystems, is of great significance for stabilizing global climate through photosynthesis and respiration (Pan et al., 2011; Griscom et al., 2017; Song et al., 2022). Meanwhile, as an indicator of ecosystem status, vegetation structure and function are highly responsive to climatic and anthropogenic influences (Li et al., 2020; Piao et al., 2020). Therefore, monitoring vegetation changes and unraveling the potential drivers are fundamental for ecosystem management and conservation.

Since ecologically fragile region is more easily influenced by climate change and anthropogenic disturbance, vegetation variations in these areas have gained increasing attention in recent years (Dardel et al., 2014; Li et al., 2021). Karst is among the most vulnerable ecosystems in the world (LeGrand, 1973). Of all karst regions worldwide, the South China Karst region is one of the three largest and most concentrated karst development areas worldwide (Lolcama, 2010), which contains eight Chinese provinces and approximately 1.9 million km^2^ in total area and carbonate rocks area of over 0.50 million km^2^. This region hosts over 200 million people and has serious shortage of cultivated land resources per capita (Jiang et al., 2014). The intense human pressures have given rise to the destruction of vegetation, intensified soil erosion and large-scale exposure of bedrocks, resulting in severe ecological degradation known as karst rocky desertification, which occupied 11.35×10^4^ km^2^ or 22% of the whole karst regions as of 2000 (Brandt et al., 2018). To reverse the expansion of rocky desertification land as well as promote vegetation cover, the central and local governments carried out many ecological engineering programs around 2000, including the Natural Forest Protection Program, Grain for Green Program, and Comprehensive Treatment of Rocky Desertification (Liao et al., 2018). Therefore, the vegetation dynamics monitoring and analysis are critical to examine the effectiveness of ecological engineering in karst areas.

The normalized difference vegetation index (NDVI) constructed by the red and near-infrared spectral bands is considered to be closely related to vegetation coverage and productivity (Huete et al., 2002), which makes it one of the most widely utilized remotely sensed indices to investigate vegetation dynamics across global and regional levels (Myneni et al., 1997; Pinzon and Tucker, 2014; Zhou et al., 2020; Lv et al., 2022). Numerous studies have employed time series of NDVI data to examine the vegetation variations in the South China Karst region since the launch of the ecological engineering around 2000 (Cai et al., 2014; Wang et al., 2015; Tong et al., 2016). These studies all used MODIS NDVI products with a resolution of 250-1000m to investigate the vegetation changes in the karst areas of South China during approximately the same time span, yet the reported vegetation change trends and spatial distribution were largely inconsistent. Differing spatial scales (i.e., pixel sizes) may be a vital reason for the inconsistency in vegetation trends (Fensholt and Proud, 2012), especially for the spatially heterogeneous South China Karst, which features staggered distribution of ground objects and discontinuous soil and vegetation cover (Zhang et al., 2017). Hence, it remains challenging to characterize actual vegetation change trends under complex terrain conditions by using coarse-resolution imagery (Vogelmann et al., 2016). Moreover, considering that ecological restoration projects started around 2000, the relatively short time-series data adopted in previous studies were impossible to characterize the vegetation changes prior to the project implementation. In this regard, effectiveness of ecological engineering on vegetation restoration may not be fully quantified due to the lack of vegetation change information before 2000 as the reference.

In recent decades, an increasing number of long time-series dense satellite images can be publicly accessed, which tremendously advances the quantification of regional and global vegetation change trends at a longer temporal scale (Zhu et al., 2016; Curtis et al., 2018; Hansen et al., 2020; Cai et al., 2022). Among these datasets is the AVHRR-based GIMMS NDVI dataset, which has also been used for vegetation trends analysis in the karst regions (Tong et al., 2017). Despite the relatively long time span (1981-2015), there remain several well-known issues in the AVHRR-derived NDVI data, such as lack of reliable onboard calibration (Teillet et al., 1997), satellite orbital drift over time (Pinzon, 2005), seasonal variations in sun-sensor viewing geometry (Fensholt and Proud, 2012), and interference by atmospheric water vapor (Liu et al., 2022). These drawbacks may cause adverse influences on the time-series observed NDVI (Nagol et al., 2014; Wang et al., 2021). Furthermore, the coarse spatial resolution of 8km for GIMMS NDVI3g data hampers its applicability in detecting vegetation dynamics taking place at local scales.

As mentioned above, the scientific community has been empowered to observe the general trend of vegetation greening in the South China Karst region at different spatial scales using dense satellite imagery (Brandt et al., 2018; Peng et al., 2021). However, it still remains disputed whether the Karst greening should be ascribed to climate change (Liu et al., 2018), human-induced ecological engineering (Qiao et al., 2021), or the joint influence of anthropogenic activities and climate variations (Wu et al., 2020). Overall, most existing studies were conducted using vegetation trend products having a coarse spatial grain or covering a short time span, thereby limiting the reliability of the attribution of vegetation dynamics. In particular, the potential influence of climate variations on vegetation dynamics using MODIS NDVI products (starting in 2000) may not be fully captured since climate change becomes more evident at long temporal ranges (Seidl et al., 2017). To date, characterization and attribution of vegetation variations covering the complete South China Karst (1.9 million km^2^) at 30m spatial scale for the past three decades remain largely understudied.

Therefore, this paper aims to characterize and attribute the vegetation dynamics covering the entire spatially heterogeneous South China Karst region for the past three decades using time series of Landsat images and the cloud-based Google Earth Engine platform. The specific research objectives are: (1) to assess spatiotemporal change patterns of vegetation in the South China Karst region at 30m spatial scale and for a notably increased time period (1987-2018), and to examine the sustainability of vegetation trends beyond 2018, (2) to further compare vegetation trends from the perspective of differing karst geological conditions, and (3) to develop a pragmatic research framework for spatially identifying the primary drivers of vegetation variations as well as quantifying their relative roles.

## 2 Materials and methods

### 2.1 Study area

**Figure 1:**
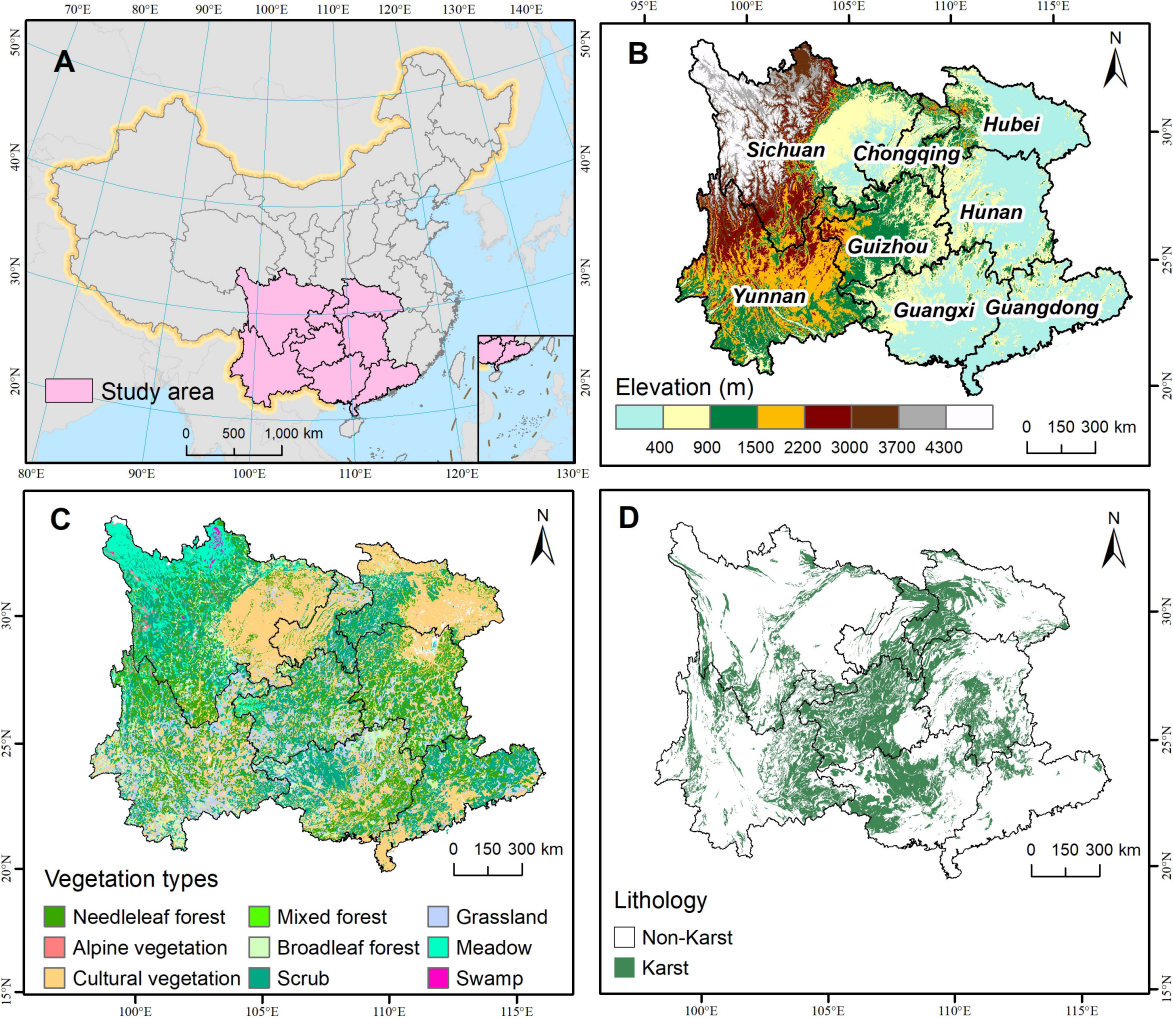
The study area. **(A)** Location of the South China Karst region in China, **(B)** elevation distribution and provincial boundaries, **(C)** spatial distribution of vegetation types and **(D)** Karst and Non-Karst region in the study area.

South China Karst region (96°50′-117°18′ E, 20°6′-34°12′ N) is among the largest and most well-developed karst concentrated areas worldwide, which covers eight Chinese provinces including Guangxi, Guizhou, Yunnan, Sichuan, Chongqing, Guangdong, Hunan and Hubei (**Figure 1**). The region occupies 1.93 million km^2^, of which 0.51 million km^2^ underlain by carbonate rocks. Besides, the study area mainly belongs to tropical/subtropical monsoon climate types, with an annual mean temperature > 15°C and annual precipitation > 1100 mm.

### 2.2 Data sources

All available surface reflectance collections provided by Landsat 5 TM, Landsat 7 ETM+ and Landsat 8 OLI images spanning from 1987 to 2018 were obtained from the Google Earth Engine (GEE) platform. All acquired Landsat imagery were atmospherically corrected using LEDAPS algorithms for Landsat 5 and 7 satellites and the LaSRC algorithms for Landsat 8 satellite. Moreover, the CFMASK algorithm was implemented to per-pixel detect and mask out snow, clouds and cloud shadows for all collected Landsat surface reflectance images (Zhu et al., 2015). In addition, the NDVI vegetation index was computed using the near infrared and red bands, and an intercept and offset were applied to Landsat 8-derived NDVI in order to compensate for the difference in sensor bands configuration between Landsat 5/7 and Landsat 8 satellites (Roy et al., 2016). Pixels with NDVI value higher than 0.90 and lower than -0.9 were masked to reduce the influence of errors like Scan Line Corrector (SLC) failure of Landsat 7 ETM+ on the final analysis (Fassnacht et al., 2019). The annual median value synthesis method was pixel-wisely employed to derive the final NDVI image for each year of the studied period (Taylor et al., 2020).

Climate data including annual average temperature and annual total precipitation during 1987-2015 were acquired from the Resource and Environment Science and Data Center (RESDC), Chinese Academy of Sciences (https://www.resdc.cn/), which were generated by the ANUSPLIN spatial interpolation method based on the daily observations from over 2400 meteorological stations in China. These two climatic factors were rasterized to the same resolution and geographical coordinate system as the counterparts of Landsat NDVI dataset. Digital elevation model (DEM) at 30m resolution was obtained from the Shuttle Radar Topography Mission (SRTM) digital elevation dataset (version 3) deposited on the GEE platform. Topographic slope and aspect were calculated by the DEM data within ArcGIS 10.2 environment. In this study, we also used lithological types vector data at the 1:500,000 scale (**Figure 1D**), which was provided by the Institute of Karst Geology, Chinese Academy of Geological Sciences (http://www.karst.cgs.gov.cn/). Land cover data for the time periods 1990 and 2018 at 1km resolution were obtained from the RESDC. Furthermore, statistical data regarding artificial afforestation area and economic investment of ecological projects for the eight provinces in South China from 2002 to 2016 were derived from the China Forestry Statistical Yearbooks.

### 2.3 Methods

#### 2.3.1 Vegetation trends quantification

In this study, the nonparametric Mann-Kendall test was adopted to assess the direction and statistical significance of the temporal trend of NDVI on the pixel scale, with the significance level set at *P*<0.05, corresponding to the test statistics |Z|>1.96. Moreover, we employed the nonparametric Theil-Sen median trend analysis to calculate the slope of the trend. Theil-Sen slope estimator mainly computes the median slopes between all n(n-1)/2 pair-wise combinations of the time-series data, which is robust and insensitive to data abnormality (Qi et al., 2019). The calculation formula is as follows:


(1) $$\text{TS=Median}\left(\frac{NDVI_{j}-NDVI_{i}}{j-i}\right),\;1987\leq i<j\leq 2018$$

where, *N* = *n* (*n* − 1)/2, *n* is the length of the time series (32 years in this study), *NDVI_i_* and *NDVI_j_* represent the NDVI values of a pixel in years *i* and *j*, respectively. *TS* is the median of the slope of *N*-pair data combination. If *TS* > 0, the time-series NDVI data show an increasing trend, and vice versa.

#### 2.3.2 Sustainability of vegetation trends in the future

The Hurst exponent, based on the rescaled range (R/S) analysis, is an effective method to reveal the future development trend of time-series data relative to the historical observations. Here we used the Hurst exponent to indicate the sustainability of vegetation dynamic trends beyond the study period (i.e., after 2018). The main calculation process is shown as follows (Sánchez Granero et al., 2008):

***Step 1: the time series {ξ(τ)} (τ = 1, 2,…, n) is divided into τ subsequence x (t), for each subsequence t = 1,…, τ***

***Step 2: define the mean sequence:***


(2) $${\langle \xi \rangle }_{\tau }=\frac{1}{\tau }\sum_{t=1}^{\tau }x(t),\;\tau =1,2,\ldots ,n$$

***Step 3: calculate the cumulative deviation:***


(3) $$X(t,\tau )=\sum_{u=1}^{t}(\xi (u)-{\langle \xi \rangle }_{\tau }),\;1\leq t\leq \tau$$

***Step 4: create a range sequence:***


(4) $$R(\tau )=\underset{1\leq t\leq \tau }{\max}X(t,\tau )-\underset{1\leq t\leq \tau }{\min}X(t,\tau ),\;\;\;\;\;\tau =1,2,\ldots ,n$$

***Step 5: create a standard deviation sequence:***


(5) $$S(\tau )=\sqrt{\frac{1}{\tau }\sum_{t=1}^{\tau }{\left(\xi (t)-{\langle \xi \rangle }_{\tau }\right)}^{2},\;\tau =1,2,\mathrm{\ldots .}n}$$

***Step 6: rescale the range:***


(6) $$\frac{R(\tau )}{S(\tau )}={\left(c\tau \right)}^{H}$$

The value of Hurst exponent (*H* value) is derived by fitting the equation log(*R/S*)*_n_* = *a* + *H* × log(*n*) based on the least squares method, which ranges from 0 to 1. When the *H* value is equal to 0.5, it conveys that the NDVI time series is a stochastic series without sustainability; When the value of *H* is greater than 0.5, it means that the time series is sustainable, and the future trend will be consistent with that during the study period; Whereas the value of *H* is less than 0.5, it indicates the anti-sustainability of the time series, namely, the trend in the future will be opposite to the counterpart during the study period.

#### 2.3.3 Relationships between vegetation variations and climatic factors

To assess the correlation between climatic variables and NDVI, partial correlation analysis was employed in this study, which measures the degree of association between two variables while excluding the influence of correlated one or one or more control variables. The calculation formula of partial correlation coefficients is as follows:


(7) $$r_{xy\cdot z}=\frac{r_{xy}-r_{xz}r_{yz}}{\sqrt{\left(1-r_{xz}^{2})(1-r_{yz}^{2}\right)}}$$

where *r_xy·z_* is the partial correlation coefficient of variable x and variable y excluding the influence of variable *z*; Variables *r_xy_*, *r_xz_* and *r_yz_* represent the simple correlation coefficients of variables *x* and *y*, *x* and *z*, *y* and *z*, respectively. The T-test method was used to validate the statistical significance of the derived partial correlation coefficient, which was set at the significance level of 0.05.

#### 2.3.4 Relative contribution of climate change and anthropogenic activities

In this study, we adopted the residual trend analysis (RESTREND) method to unravel the relative contribution of climate change and human activities to vegetation changes (Peng et al., 2021). The general principle of the RESTREND method is to establish a multivariate regression model between NDVI and climatic variables, and then derive the NDVI residuals between observed NDVI (*NDVI_obs_*) and predicted NDVI (*NDVI_pre_*) driven by the resultant NDVI-climate model. The change trend of NDVI residuals over time can be attributed to the impact of anthropogenic activities. The specific calculation procedures are shown as follows:

Firstly, we developed a multivariate regression model between NDVI (response variable) and climate factors (temperature and precipitation as explanatory variables). To better reflect the effects of ecological engineering on vegetation change, the NDVI-climate model was constructed during the reference period without project influences, i.e., before the implementation of ecological engineering (1987-1999) instead of the full period (Tong et al., 2017). The calculation formula is as follows:


(8) $$NDVI_{pre}(i,t)=a*Temp(i,t)+b*Prec(i,t)+c$$

where *i* is the location of the pixel, *t* is the year, *a* is the regression coefficient between NDVI and annual average temperature (*Temp*), *b* is the regression coefficient between NDVI and annual precipitation (*Prec*), and *c* is a constant term.

Subsequently, based on the established regression model for the reference period, we calculated the predicted NDVI over 2000 to 2015 on the pixel scale, which was considered as the NDVI driven by climate change alone. Then, we computed the residual difference between observed NDVI (*NDVI_obs_*) and predicted NDVI (*NDVI_pre_*), and obtained the time series NDVI residuals (*NDVI_res_*), which were expected to reflect the vegetation trend driven by human activities. The calculation formula of residual difference is as follows:


(9) $$NDVI_{res}(i,t)=NDVI_{obs}(i,t)-NDVI_{pre}(i,t)$$

where *NDVI_res_* (*i*, *t*), *NDVI_obs_* (*i*, *t*) and *NDVI_pre_* (*i*, *t*) represent the NDVI residual, observed NDVI and predicted NDVI values simulated by climate change of pixel *i* in year *t*, respectively.

In this study, the effect of climate change on vegetation variations was represented by the trends of *NDVI_pre_*, whereas the effect of human activities was measured by the trends of *NDVI_res_*. To discern the climatic and anthropogenic influences on vegetation change, we calculated the trends of *NDVI_pre_*, *NDVI_obs_* and *NDVI_res_* using the Theil-Sen slope estimator. Finally, the relative importance of climate change and human activities to vegetation dynamics was determined on the basis of *Slope(NDVI_obs_)*, *Slope(NDVI_pre_)* and *Slope(NDVI_res_)* as shown in **Table 1** (Qi et al., 2019; Li et al., 2021).

**Table 1:** Identification method and contribution quantification of the drivers for vegetation changes under differing scenarios.

		Slope(NDVI_pre_)	Slope(NDVI_res_)	Relative contribution of climate change (%)	Relative contribution of human activities (%)	Attribution
The zone of vegetation recovery	Scenario 1	>0	>0	$\frac{Slope{\left(NDVI\right)}_{pre}}{Slope{\left(NDVI\right)}_{obs}}\times 100$	$\frac{Slope{\left(NDVI\right)}_{res}}{Slope{\left(NDVI\right)}_{obs}}\times 100$	Both climate change and human activities contributed to vegetation recovery
	Scenario 2	>0	<0	100	0	Climate-induced vegetation recovery
	Scenario 3	<0	>0	0	100	Human-induced vegetation recovery
The zone of vegetation decrease	Scenario 1	<0	<0	$\frac{Slope{\left(NDVI\right)}_{pre}}{Slope{\left(NDVI\right)}_{obs}}\times 100$	$\frac{Slope{\left(NDVI\right)}_{res}}{Slope{\left(NDVI\right)}_{obs}}\times 100$	Both climate change and human activities induced the vegetation decrease
	Scenario 2	<0	>0	100	0	Climate-induced vegetation decrease
	Scenario 3	>0	<0	0	100	Human-induced vegetation decrease

## 3 Results

### 3.1 Vegetation dynamics and sustainability assessment

#### 3.1.1 Temporal variations of NDVI from 1987 to 2018

**Figure 2:**
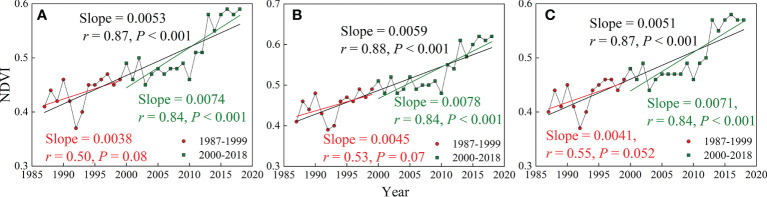
Interannual variations of NDVI from 1987 to 2018 in **(A)** southern China, **(B)** karst region, and **(C)** non-karst region. Note that the black solid line is the linear fitting of NDVI for the full time series (1987-2018), while the red and green solid line denote the linear fitting of NDVI for the reference period (1987-1999) and conservation period (2000-2018), respectively.

The interannual changes in NDVI indicated a significantly increasing trend of vegetation cover in southern China during 1987-2018 (**Figure 2A**), with an increase rate of 0.0053 yr^-1^ (*r* = 0.87, *P*<0.001). Moreover, it is found that during the conservation period (2000-2018), the annual increase rate of NDVI (0.0074 yr^-1^) has nearly doubled in contrast with the counterpart over the reference period (1987-1999). This indicates that vegetation growth has been substantially improved after the establishment of ecological restoration projects. In addition, the discrepancy in NDVI temporal change under different geological backgrounds has also been investigated (**Figures 2B, C**). The annual increase rate of NDVI in karst areas (0.0059 yr^-1^) was greater than that in non-karst areas (0.0051 yr^-1^), which suggested the vegetation restored more rapidly in karst areas compared with non-karst areas.

#### 3.1.2 Spatial differentiation of vegetation trends during 1987-2018

**Figure 3:**
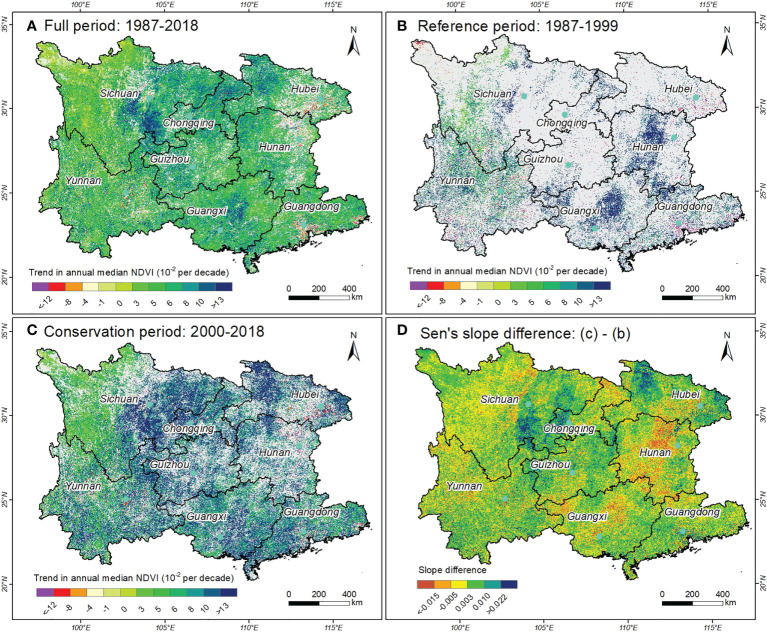
Spatial distribution patterns of NDVI trends for different periods in southern China. **(A)** Full time series (1987-2018), **(B)** reference period (1987-1999), **(C)** conservation period (2000-2018), and **(D)** difference in Sen’s slope of vegetation trends calculated by Theil-Sen slope estimator between the conservation period and reference period. Note that the grey area denotes not statistically significant (*P* > 0.05), and the bright green solid dot denotes the location of the capital cities of eight provinces in southern China.

**Figure 3A** shows the variation trend of annual NDVI over the past three decades on the pixel scale. Specifically, the proportion of pixels with significant (*P<* 0.05) increase in vegetation cover reached 74.80% in southern China, which was primarily contributed by forest ecosystems (55%), followed by croplands (24%) and grasslands (19%). In contrast, only 2.18% of the study area presented a significantly decreasing trend, which was largely concentrated in urban clusters and their surrounding areas, such as the Pearl River Delta of Guangdong and Wuhan urban agglomeration of Hubei.

Furthermore, we compared the difference of vegetation change trends prior to and after the implementation of ecological restoration projects (**Figures 3B, C**). Notably, after the implementation of the projects (2000-2018), the proportion of pixels with significant vegetation restoration increased to 57.45%, more than triple the amount before the project implementation (17.17%). Moreover, only 1.93% of the study area presented a significant downtrend in vegetation cover, which was slightly higher than that before the project implementation (1.64%). The slope difference between the two periods also revealed evident spatial heterogeneity (**Figure 3D**). Regions where the NDVI slope over the conservation period was larger than that prior to 2000 accounted for 62.86% of the study area, implying that accelerated vegetation restoration has been observed in the most areas of South China Karst region.

#### 3.1.3 Sustainability of vegetation trends beyond 2018

The future vegetation trends after 2018 was also examined based on the rescaled range (R/S) analysis (**Supplementary Figure S1**). The mean Hurst exponent of NDVI in southern China was 0.661, indicating that the vegetation change trend is sustainable in the South China Karst region as a whole. Spatially, pixels with Hurst exponent exceeding 0.5 accounted for 93.30% of the study area (**Supplementary Figure S1A**). The spatial pattern of future vegetation trends based on vegetation dynamics (1987-2018) and Hurst exponent was shown in **Supplementary Figure S1B**. The majority (72.22%) of the total area revealed a sustained uptrend in vegetation cover beyond 2018 (**Table 2**). Additionally, it is found that the area proportion of persistent vegetation improvement in karst areas (74.12%) was higher than that in non-karst areas (71.55%). Meanwhile, the area proportion of persistent vegetation degradation in karst areas (1.42%) is lower than the counterpart in non-karst areas (2.41%). These findings highlighted comparatively more sustainable increasing vegetation trends in karst areas in contrast with non-karst areas.

**Table 2:** Area ratio of different future vegetation change trend types based on the Theil-Sen trend slope (*TS_NDVI_*), Mann-Kendall test (|*Z*|) and Hurst exponent (*H*).

Code	Future trends	*TS_NDVI_*	|*Z*|	*H*	Area proportion (%)		
					Southern China	Karst areas	Non-karst areas
1	Uncertain	—	≤1.96	[0, 0.5]	4.08	4.40	3.97
2	Decrease to increase	<0	>1.96	[0, 0.5]	0.03	0.02	0.04
3	Increase to decrease	>0	>1.96	[0, 0.5]	2.58	2.48	2.62
4	Persistent stable	—	≤1.96	(0.5, 1]	18.93	17.55	19.42
5	Persistent decrease	<0	>1.96	(0.5, 1]	2.15	1.42	2.41
6	Persistent increase	>0	>1.96	(0.5, 1]	72.22	74.12	71.55

Note that the specific properties of the numeric code of the six future vegetation trend types in **Supplementary Figure S1** were consistent with the counterparts in **Table 2**.

### 3.2 Comparative analysis of vegetation dynamics in different geological regions

The multi-year average NDVI in the karst areas was larger than the counterparts in non-karst areas during all three time periods (**Supplementary Figure S2A**), suggesting that karst regions had relatively higher vegetation cover in comparison with non-karst areas. Regarding the vegetation dynamic trends, karst areas shared comparatively larger area ratio of significant vegetation increase (76.61%) than that in non-karst regions (74.21%) over the period 1987-2018 (**Supplementary Figure S2B**). This indicated that vegetation restoration was more evident in karst areas. If divided into two periods, prior to the launch of ecological engineering (1987-1999), the majority of pixels showed no significant changes for both karst and non-karst regions. Conversely, after the project implementation (2000-2018), the vegetation change was dominated by significant uptrends in both geological regions.

### 3.3 Attribution of vegetation dynamics

#### 3.3.1 Correlations between climate change and vegetation dynamics

To quantify the influence of climate change on vegetation variations, we calculated the partial correlation coefficients between climatic factors and NDVI at the pixel scale, which showed evident spatial differences (**Supplementary Figure S3**). Specifically, regions where temperature and NDVI were significantly (*P*< 0.05) positively correlated covered 21.38% of the whole area. These regions were also the areas with partial correlation coefficient greater than 0.5, mostly located in Yunnan province and Northwest Sichuan Plateau. By comparison, the proportion of the areas where the temperature had significant negative relationship with NDVI attained only 2.15%, which was largely distributed in the Pearl River Delta and the coastal areas of western Guangdong. Consequently, up to 76.47% of regions had no significant correlations between annual temperature and NDVI.

Moreover, the partial correlation coefficients between annual precipitation and NDVI also revealed apparent regional differentiation (**Supplementary Figures S3C, D**). Regions where annual precipitation and NDVI presented a significant positive correlation accounted for only 3.75%. Geographically, it was mostly concentrated in southern Guangxi and northern Guangdong, and the partial correlation coefficient was larger than 0.5. In addition, regions where annual precipitation was significantly negatively correlated with NDVI were slightly higher in proportion, accounting for 5.91%, which was largely concentrated in northwest Yunnan and southwest Sichuan. As a consequence, 90.34% of the areas had no significant relationships between annual precipitation and NDVI. These findings suggest that, in spite of the warmer and drier climate change trend during the past three decades (**Supplementary Figure S4****,** **S5**), climatic factors have no significant impacts on vegetation change in most areas of southern China, implying that non-climate variables contributed largely to vegetation recovery in the South China Karst.

#### 3.3.2 Relative contribution of climate change and anthropogenic activities

**Figure 4:**
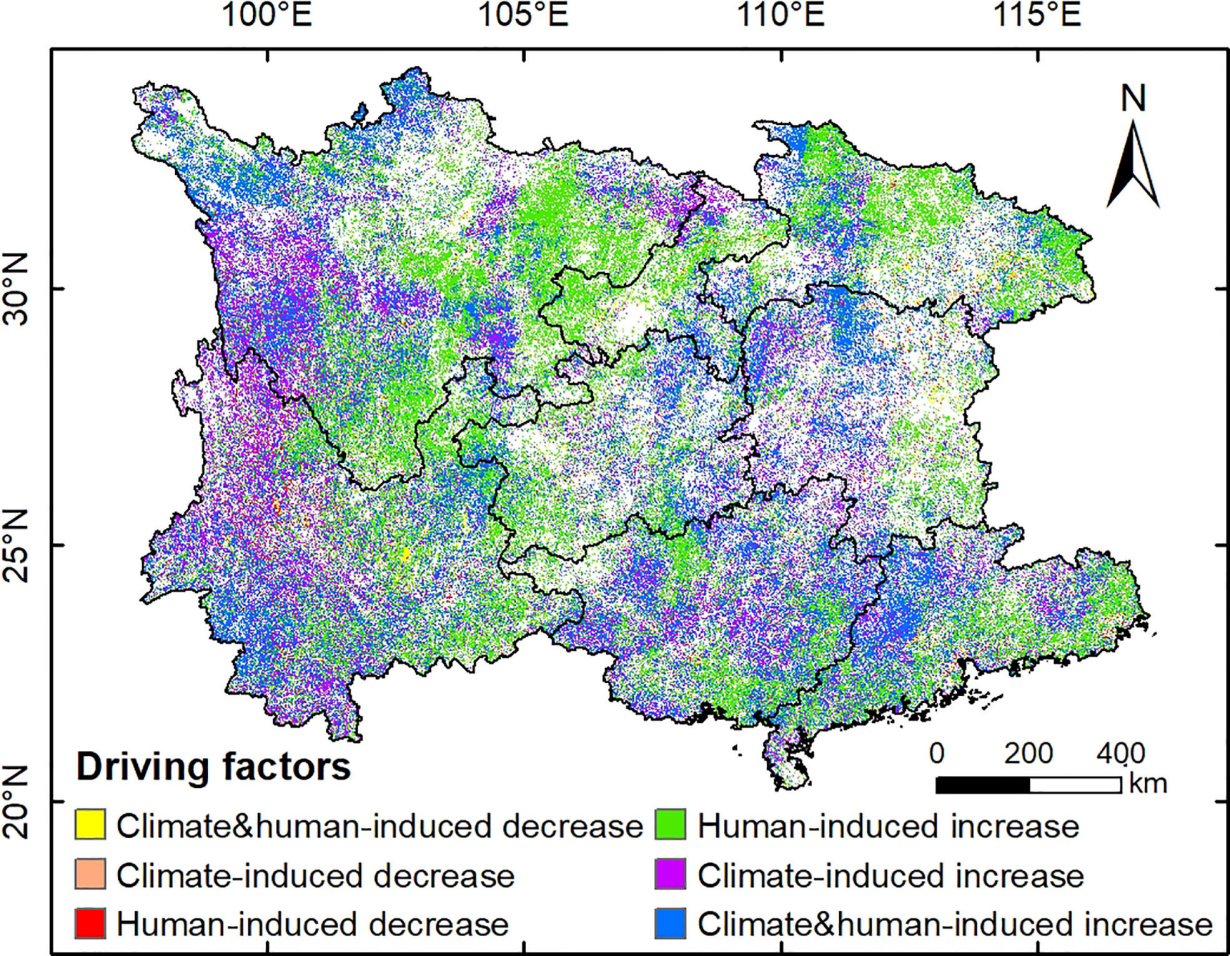
Spatial distribution of driving factors for vegetation variations in the regions with significant NDVI change (*P*< 0.05) in southern China.

In this study, we identified the driving factors of vegetation change on the pixel scale using multivariate regression and residual trend analysis method (**Figure 4**). Analysis showed that the significant increasing vegetation trends led to anthropogenic influences standalone occupied 40.75% of the total area of significant vegetation increase, principally located in the eastern Sichuan Basin, mountain areas of southwestern Sichuan, northern and eastern Hubei. Contrastingly, climatic factors alone contributed only 21.37% to significant vegetation increase, which was largely distributed in the southwestern Sichuan and northwestern Yunnan. This indicated that the relative importance of anthropogenic activities to vegetation restoration was far greater than the counterpart of climate change in the South China Karst. In addition, the area of vegetation increase caused by the joint influence of climate change and anthropogenic activities covered 37.88% of the whole area of significant vegetation increase, which was found scattered in the north of Northwest Sichuan Plateau, southwest Yunnan, north Hunan and northwest Guangdong. In contrast, the areal proportion of human-induced vegetation decrease was rather trivial, covering only 1.56% of the areas with significant vegetation changes, which were primarily distributed in the highly developed urban areas and their surrounding regions, such as the Pearl River Delta region in Guangdong province. Similarly, the proportion of climate and human-induced vegetation decrease was also small (1.48%), and areas showing vegetation decrease caused by climate change alone covered only 0.16%.

**Figure 5:**
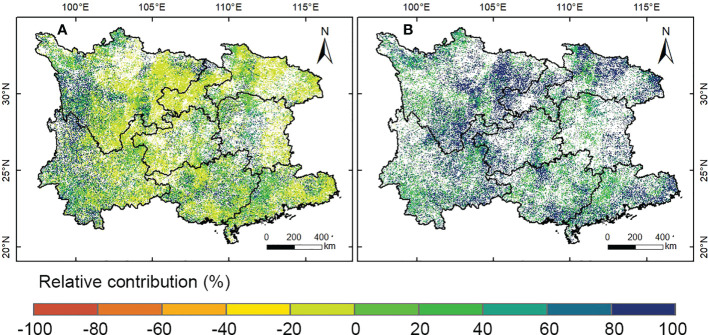
Spatial distribution of relative contribution of climate change and human activities in regions with significant NDVI change (*P*< 0.05). **(A)** Climate change, and **(B)** human activities.

To quantitatively disaggregate the climatic and anthropogenic effects on vegetation dynamics in the South China Karst region, we further investigated the relative contribution of climate change and human activities to vegetation dynamics on the pixel scale (30m) (**Figure 5**). Specifically, the relative role of climate change to vegetation variations was mostly concentrated within 20%, accounting for 70.59% of the total area with significant NDVI change (*P*< 0.05), indicating that climate change imposed a minor effect on vegetation variations in most areas. Whereas areal proportion of regions with climate change contributing more than 80% to vegetation increase achieved only 19.73%, which was primarily located in the south of Northwest Sichuan Plateau, northwest and southwest Yunnan. By comparison, the regions with relative contribution of anthropogenic activities to vegetation increase exceeding 80% covered 52.67% of the areas with significant vegetation change, far larger than the counterpart of climate change. These regions were widely distributed in the eastern Sichuan, eastern and northern Hubei, southern Guangxi and eastern Guangdong. This indicates that anthropogenic activities are the dominant driver for the significant vegetation recovery in most areas of the South China Karst region, which is largely attributable to the implementation of large-scale ecological engineering.

**Figure 6:**
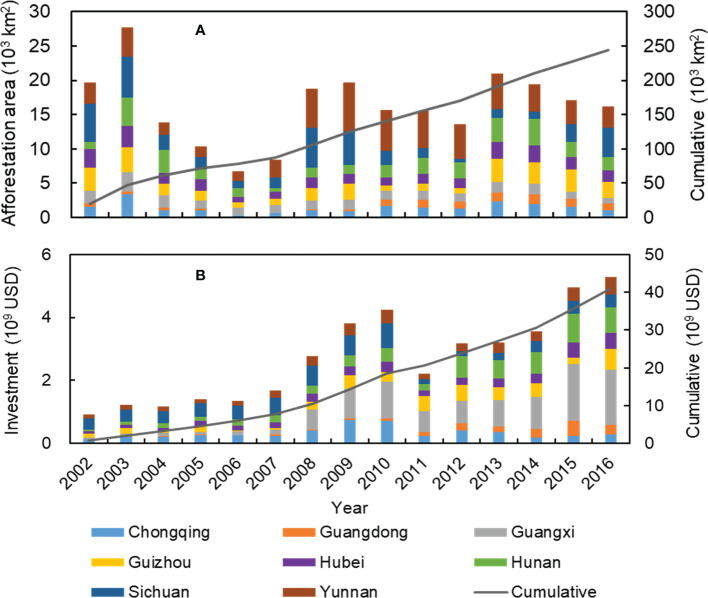
Annual (left y-axis) and cumulative (right y-axis) afforestation area and forestry investment in the eight provinces in southern China from 2002 to 2016. **(A)** Afforestation area, and **(B)** forestry investment.

Statistical data showed that from 2002 to 2016, the Chinese government has invested 40.94 billion USD in total into the afforestation projects (**Figure 6**), and more than 16,000 km^2^ of trees has been planted annually in southern China. Analysis showed that there was a significant (*P*< 0.05) positive relationship between annual NDVI and afforestation area in the southern China as a whole and its eight provinces (**Supplementary Figure S6**). This implies that the afforestation projects significantly enhanced the increase of regional vegetation cover.

## 4 Discussion

### 4.1 Spatial-temporal changes of vegetation greenness

This study constructed a three decadal vegetation index dataset at 30m spatial resolution covering the complete South China Karst region using time-series Landsat images, and obtained fine-scale information on vegetation dynamics during 1987-2018. In accordance with other research, our study also revealed an extensive vegetation recovery in the vegetation across the South China Karst during the past three decades (Macias-Fauria, 2018; Peng et al., 2021). Our result showed that the proportion of pixels with significant vegetation greening reached 74.80% from 1987 to 2018 using Landsat-based NDVI data (**Figure 3A**), significantly higher than the counterpart detected by the GIMMS NDVI3g data used by previous research (Tong et al., 2017; Zhao et al., 2020; Zhang et al., 2021). It is noteworthy that these studies all applied long time-series GIMMS NDVI3g dataset at 8km resolution. Generally speaking, the larger the pixel, the more likely it is to contain differing land cover types, that is, the more significant the mixed pixel effect (Hansen and Loveland, 2012). Especially for karst areas, small-scale vegetation change is particularly common due to the complex mosaic landscapes and the resulting high spatial heterogeneity in this region (Zhang et al., 2017). Hence, coarse spatial resolution greatly limits the response range of vegetation change that can be detected (Vogelmann et al., 2016; Huang et al., 2020). For instance, Wang et al. (2021) found that GIMMS NDVI data underestimated the changes of alpine vegetation growth over the past 30 years based on long-term field observations. Notably, our study found that there were much more pixels (57.45%) showing vegetation greening during the conservation period (2000-2018) in contrast to the reference period (17.17%) during 1987-1999. This indicates that the vegetation restoration has accelerated in the South China Karst ensuing the practice of ecological projects starting around 2000 (Chen et al., 2021). Similar phenomenon has also been reported in the Chinese Loess Plateau where the Grain for Green project has been largely established (Sun et al., 2015).

Moreover, we found that there existed a discrepancy in vegetation dynamics under different geological conditions. To be specific, from the perspective of interannual variations, a higher increase rate of vegetation greenness was observed in karst regions in contrast with non-karst regions (**Figure 2**). In addition, more pixels showing significant vegetation uptrend were detected in karst regions (**Supplementary Figure S2B**). It can be seen that vegetation restoration is more rapid and takes place in larger areal proportions in the carbonate rock areas. From the perspective of material circulation, rocks are the main source of soil minerals. Because the types and contents of minerals contained in rocks of different lithology are different, the contents of nutrient elements provided by rocks vary greatly, so lithology has a great impact on soil fertility (Wang et al., 2004). The physical and chemical properties of soil play a key role in the vegetation growth and its change trends, so lithology is an important natural factor influencing the growth and change of vegetation (Qiao et al., 2020). Hence, when implementing ecological engineering in the future, it is necessary consider the temporal and spatial differences of vegetation restoration under different lithological conditions, and take ecological construction and protection measures according to local conditions.

### 4.2 Effects of climate change on vegetation dynamics

Numerous research has shown that climate change is one of the leading drivers of vegetation variations in China (Zhu et al., 2016; Jiang et al., 2022). However, our study illustrated that the effects of climate change on vegetation dynamics in South China Karst were limited as jointly demonstrated by the partial correlation analysis (**Supplementary Figure S3**) and residual trend analysis (**Figure 5A**). This accords with previous research (Tong et al., 2016; Qiao et al., 2021). Moreover, the relationship between NDVI and precipitation is even weaker than that between NDVI and temperature. This may be because the South China Karst mainly locates in the subtropical monsoon climate zone, with abundant rainfalls and moderate temperature (Liu et al., 2015), and the vegetation growth is not responsive to slight changes in rainfalls (Piao et al., 2020). This is different from arid and semi-arid areas where precipitation is the primary controlling environmental variable affecting vegetation growth (Sun et al., 2015), and increased precipitation promotes vegetation cover in these areas. Furthermore, affected by the special karst geological background, the South China Karst region has a two-dimensional hydrological structure of surface water and groundwater, which are well linked *via* fractures as well as sinkholes (Wang et al., 2019). Despite abundant rainfall in the growing season, surface runoff still flows into the groundwater system through karst fissures, pits and rivers (Zhang et al., 2017), which implies that the rainfalls in the South China Karst cannot be fully utilized for the growth of vegetation. Meanwhile, karst soil mainly comes from the residue left after the dissolution of parent carbonate rock (Wang et al., 1999). Due to the slow soil forming rate of carbonate rocks, the soil layer in the region is generally thin and the soil cover is scarce and discontinuous, especially in rocky desertification area (Jiang et al., 2014). Although the annual precipitation in the region is relatively high, the soil water holding capacity is weak. This leads to low soil humidity in karst areas, which is not enough to meet the surface evapotranspiration demand in the region and may give rise to the occurrence of extreme droughts (Liu et al., 2011).

In this study, we found an apparent warming and drying climate trend in the South China Karst region over the three decades (**Supplementary Figures S4****,** **S5**), especially after 2000, which is in accordance with existing reports (Lian et al., 2015). Although the increase of temperature will prolong the length of vegetation growing season and increase the intensity of photosynthetic rate (Dragoni et al., 2011), the sustained temperature increase may further accelerate the plants transpiration and surface evapotranspiration (Barriopedro et al., 2012; Jia et al., 2022). Meanwhile, the decrease of precipitation further promotes the decline of soil moisture in karst areas (Liu et al., 2018), which may inhibit the growth of vegetation to a certain extent. Although the trend of climate change may be unfavorable to vegetation growth after 2000, vegetation restoration trend during this period is more obvious than that prior to 2000, which once again confirms that climate change is not the primary factor for vegetation restoration in the South China Karst.

However, extreme drought climate poses a serious threat to the future development of vegetation (Li et al., 2019). Several studies have pointed out that for the past half century, the intensity and duration of seasonal drought events in South China have also increased coupled with the increase of temperature and decline of precipitation (Han et al., 2014; Yu et al., 2014). In particular, southwest China suffered extreme drought rarely seen in a century from 2009 to 2010, which directly led to the decline of regional vegetation productivity, the increase of tree mortality and the significant destruction of vegetation (Barriopedro et al., 2012). Due to the occurrence of rocky desertification, the frequency of extreme drought in karst areas has increased in recent decades (Jiang et al., 2014). Different studies show that large-scale drought has significantly reduced vegetation activities through different research approaches such as experimental methods, satellite observations and dynamic vegetation models (Zhao and Running, 2010). Considering that the duration and frequency of drought are increasing, it may offset the effectiveness of ecological restoration projects on vegetation increase (Wu et al., 2014). In view of this, under the future climate change scenarios, special attention should be paid to take more targeted measures to counter the negative impact of extreme climate on vegetation growth. For example, it is suggested to select more drought resistant tree species in future afforestation projects to improve rainwater utilization efficiency.

### 4.3 Anthropogenic influence on vegetation dynamics

In this study, we quantified the relative role of anthropogenic activities to vegetation variations at a pixel size of 30m in the South China Karst region, and found that the vast majority of the significant vegetation recovery was dominated by anthropogenic influences (**Figure 5**). Consistent with our results, increasing studies have also highlighted the significant positive impact of human activities, especially large-scale afforestation, on the increase of vegetation cover in karst areas (Viña et al., 2016; Zhao et al., 2020). In addition, analysis indicated a significant, positive association between NDVI and afforestation area at the provincial level (**Supplementary Figure S6**), implying that ecological projects would enhance vegetation cover in the region, which accords with existing research (Niu et al., 2019). Nevertheless, there are still some deficiencies in the design of the existing ecological restoration projects, which need to be improved in the follow-up project planning. For instance, a limited number of fast-growing tree species has been prioritized in current conservation measures, resulting in poor biodiversity and vulnerability to insect damage or fire risks (Hua et al., 2016).

Despite the positive effects of ecological projects, anthropogenic interferences such as urban expansion, deforestation and reclamation could also impose adverse effects on vegetation growth (Song et al., 2018). Our study revealed that significant vegetation decrease was mostly observed in urban agglomerations such as Pearl River Delta region of Guangdong province and Wuhan Urban Agglomeration of Hubei province (**Figure 3**). Further, based on the NDVI change trend during 1987-2018 and the corresponding land use changes over the same period (**Supplementary Figure S7**), we found that the areas with significant vegetation degradation are highly consistent with the regions experiencing urban expansion in the past three decades. In addition, although local farmers can obtain certain economic compensation by participating in the Grain for Green Program (Liang et al., 2012), the income from crops cultivation and agricultural subsidies exceeds the compensation standard for ecological projects, resulting in deforestation and steeply sloped cultivation in some regions. This may partly interpret the vegetation browning detected in the region.

### 4.4 Limitations and future work

Overall, this study provides an easy-to-use research framework for detecting and attributing vegetation changes in highly fragmentized landscapes at large spatial spans while with comparably high spatial resolution. However, there are still several issues that need to be resolved in future research. Firstly, this study quantified the partial correlation between NDVI and climatic factors on the interannual scale, without considering the time-lag effect of climatic factors on vegetation growth (Saatchi et al., 2013; Wu et al., 2015). Secondly, previous studies have shown that vegetation change is affected by many other factors, such as nitrogen deposition and atmospheric CO_2_ concentration (Piao et al., 2020). Nevertheless, due to the lack of long-term spatial data, the influences of nitrogen deposition and CO_2_ fertilization on karst vegetation growth have not been considered, which may further increase the uncertainty of the research results. Moreover, we derived NDVI residual sequence by eliminating the influence of climate variability based on the constructed NDVI-climate model, and assumed that NDVI residuals were mainly caused by anthropogenic factors. However, extreme events such as wildfires and droughts may also influence vegetation change (Song et al., 2019), whereas their effects are not yet considered in the RESTREND method, which might add to the uncertainty of the study.

## 5 Conclusions

This study examined the spatial and temporal variability of vegetation greenness at a spatial resolution of 30m for the period 1987-2018 across the entire South China Karst using time-series Landsat-based NDVI and the Google Earth Engine cloud platform, which helps to resolve the contradiction between high spatial heterogeneity in the karst landscapes and low spatial resolution remote sensing monitoring as practiced by previous studies. The main conclusions were summarized as follows:

1. This study confirmed an extensive vegetation recovery in the South China Karst during the past three decades, consistent with existing studies but with a higher greenery proportion (74.80%) revealed by finer-resolution satellite observations.
2. The area of vegetation recovery tripled after the implementation of ecological engineering compared with the reference period (1987-1999).
3. The majority of the vegetation restoration will be sustainable after the study period as indicated by the Hurst exponent.
4. The climate in the region tended to be warmer and drier, whereas climate change imposed limited impacts on vegetation variations.
5. Human-induced ecological engineering starting around 2000 dominated the vegetation recovery in most areas.

## Data availability statement

The original contributions presented in the study are included in the article/**Supplementary Material**. Further inquiries can be directed to the corresponding author.

## Author contributions

JP, LiW and ZN conceived the study and wrote the manuscript. JP, HH, LeiW, XW, WL, HY, JC and HF conducted formal analysis. All authors contributed to the article and approved the submitted version.

## Funding

This study was funded by the National Key Research and Development Program of China (No. 2021YFE0117900), the Guangdong Basic and Applied Basic Research Foundation (Nos. 2020A1515110172, 2021A1515110442), Jiangxi Provincial Science and Technology Special Project of Jinggangshan Agricultural High-tech Industrial Demonstration Zone, the National Natural Science Foundation of China (Nos. 41871347, 42171369), the Foundation of President of the Zhongke-Ji’an Institute for Eco-Environmental Sciences (Nos. ZJIEES-2021-01, ZJIEES-2022-02), and the Science and Technology Project of Jinggangshan Agricultural High-tech Industrial Demonstration Zone (No. 202151).

## Conflict of interest

The authors declare that the research was conducted in the absence of any commercial or financial relationships that could be construed as a potential conflict of interest.

## Publisher’s note

All claims expressed in this article are solely those of the authors and do not necessarily represent those of their affiliated organizations, or those of the publisher, the editors and the reviewers. Any product that may be evaluated in this article, or claim that may be made by its manufacturer, is not guaranteed or endorsed by the publisher.
