# Supplementary material for: Characterization and attribution of vegetation dynamics in the ecologically fragile South China Karst: Evidence from three decadal Landsat observations
Source: Front Plant Sci. 2022 Oct 27;13:1043389. doi: 10.3389/fpls.2022.1043389 (PMC9648820; doi:10.3389/fpls.2022.1043389)
Supplement: Supplementary file 1 [file DataSheet_1.pdf]

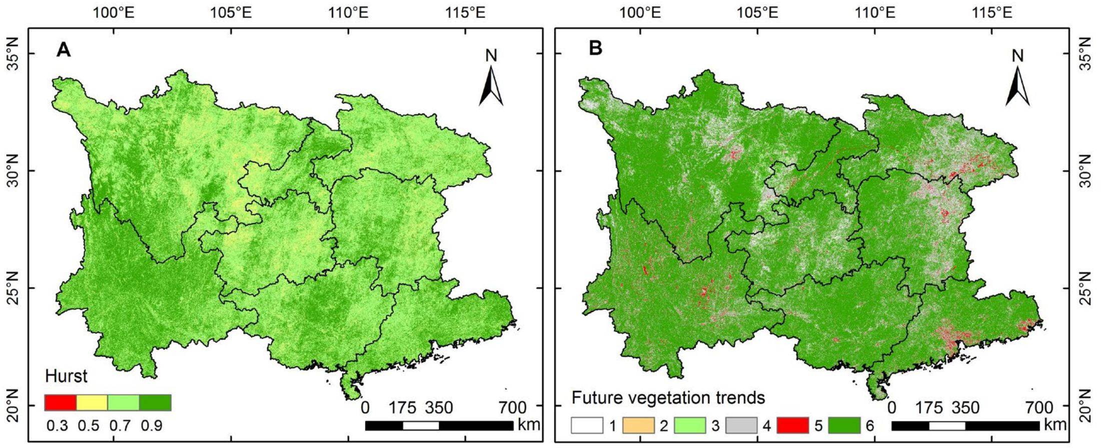

Supplementary Figure S1. Spatial distribution of the (A) Hurst exponent and (B) future vegetation trends based on observed vegetation dynamics (1987-2018) in southern China. Note that the specific properties of the numeric code of the six future vegetation trend types were depicted in Table 2.

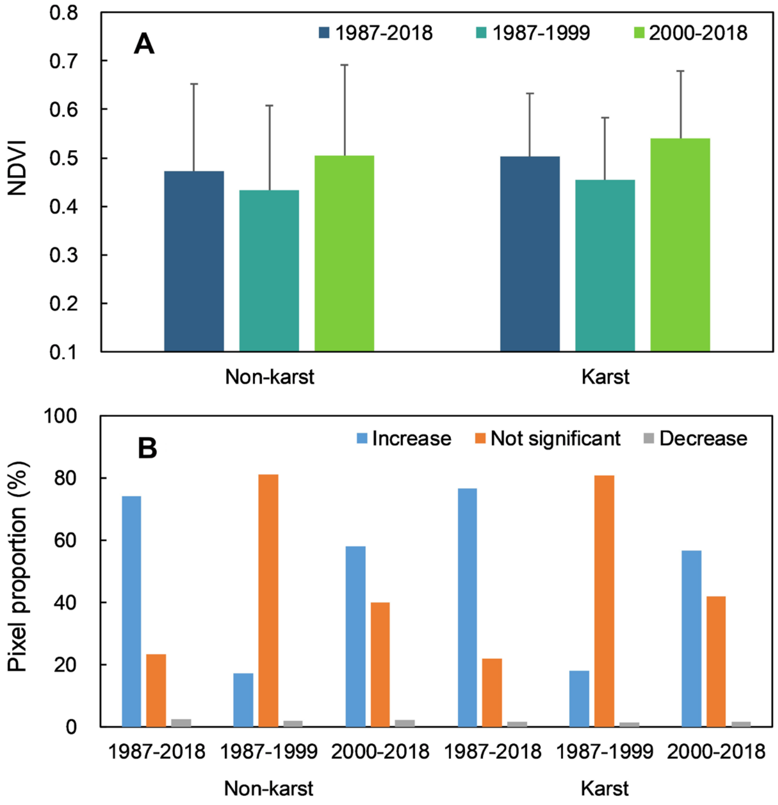

Supplementary Figure S2. Comparison of the (A) multi-year average NDVI, and (B) pixel proportion of various vegetation trend types in karst and non-karst regions over different periods.

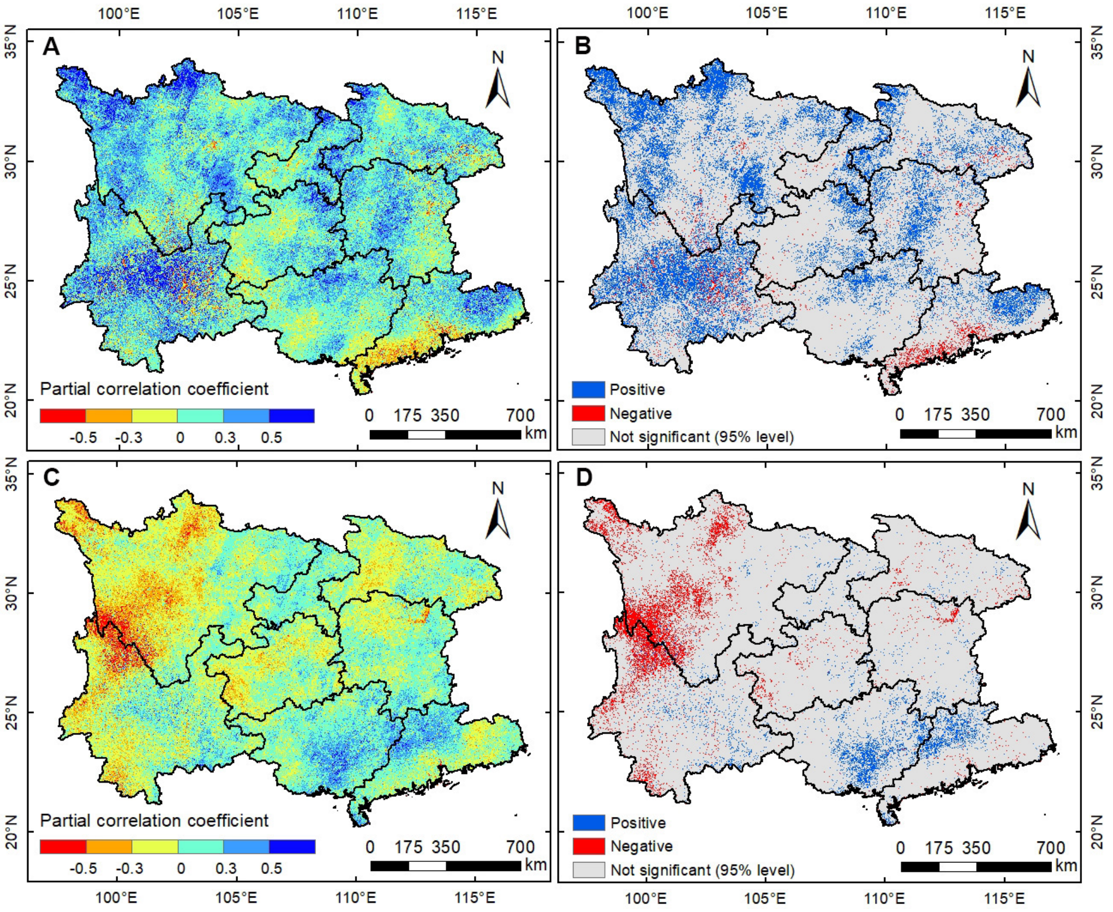

Supplementary Figure S3. Spatial distribution of partial correlation coefficients between NDVI and climatic factors. (A) Annual mean temperature vesus NDVI and (B) its corresponding statistical significance, (C) annual total precipitation vesus NDVI and (D) its corresponding statistical significance. The significance level was determined at $P < 0.05$ in this study.

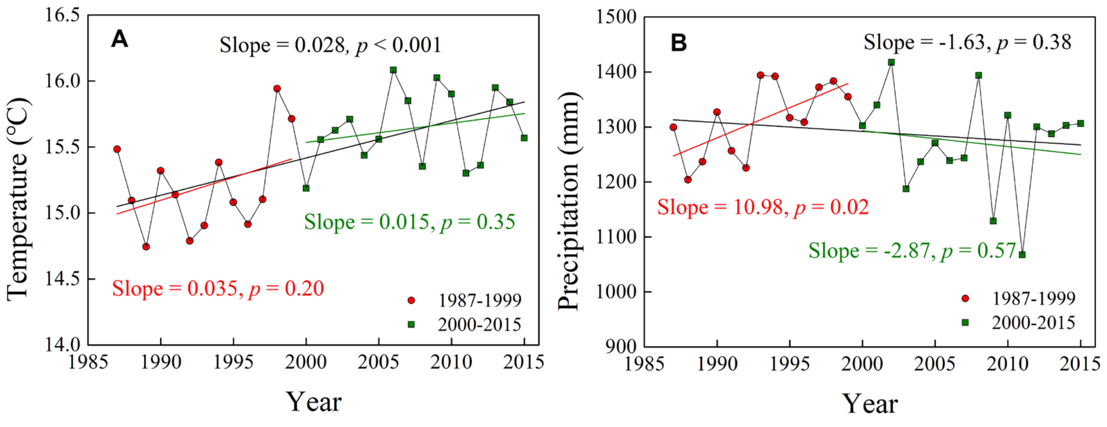

Supplementary Figure S4. Interannual variations of climatic factors in southern China from 1987 to 2015. (A) Annual mean temperature, and (B) annual total precipitation.

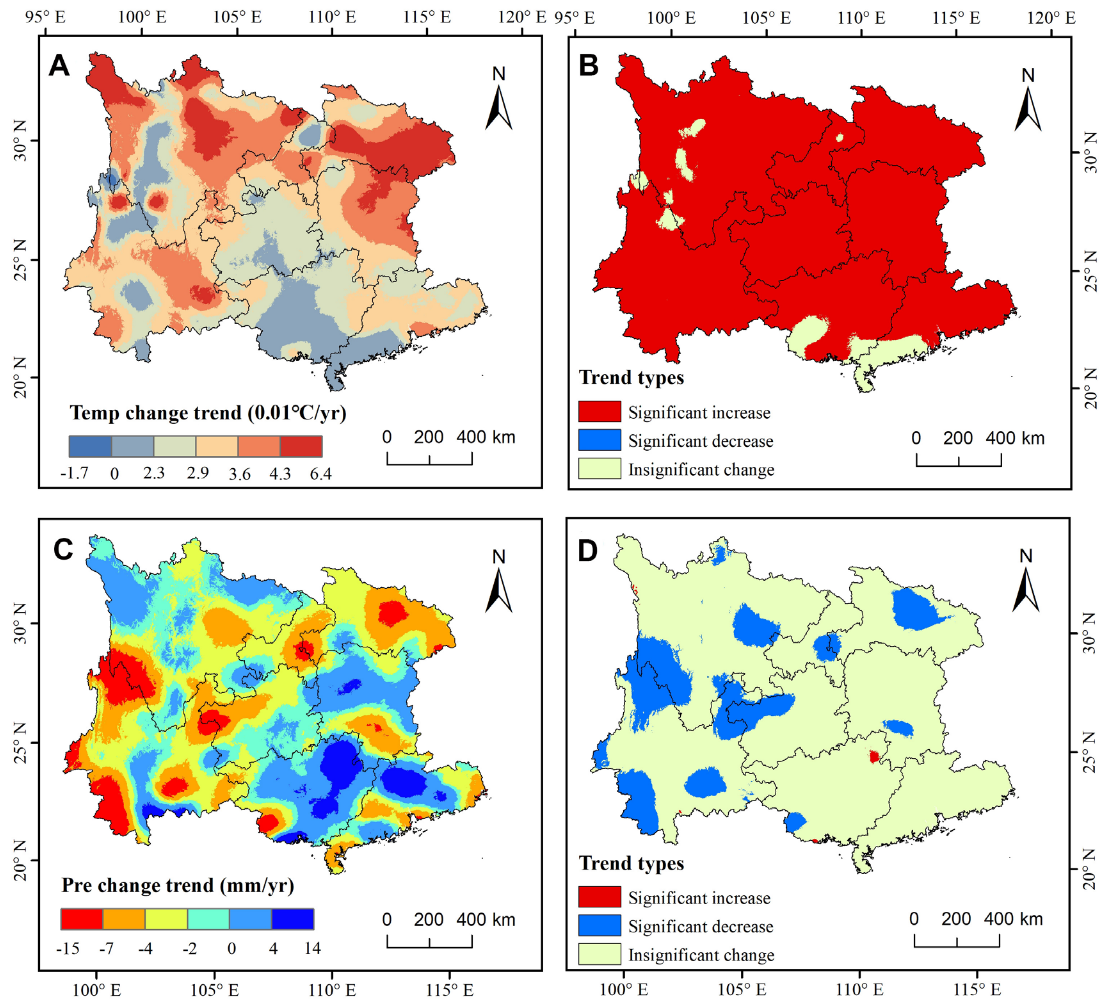

Supplementary Figure S5. Climate change rate and trend types in the spatial domain in southern China during the past three decades. Change trend for (A) annual mean temperature (temp) and (C) annual precipitation (pre); *P*-values of (B) temperature change trend and (D) precipitation change trend. The significance level was determined at $P < 0.05$ in this study.

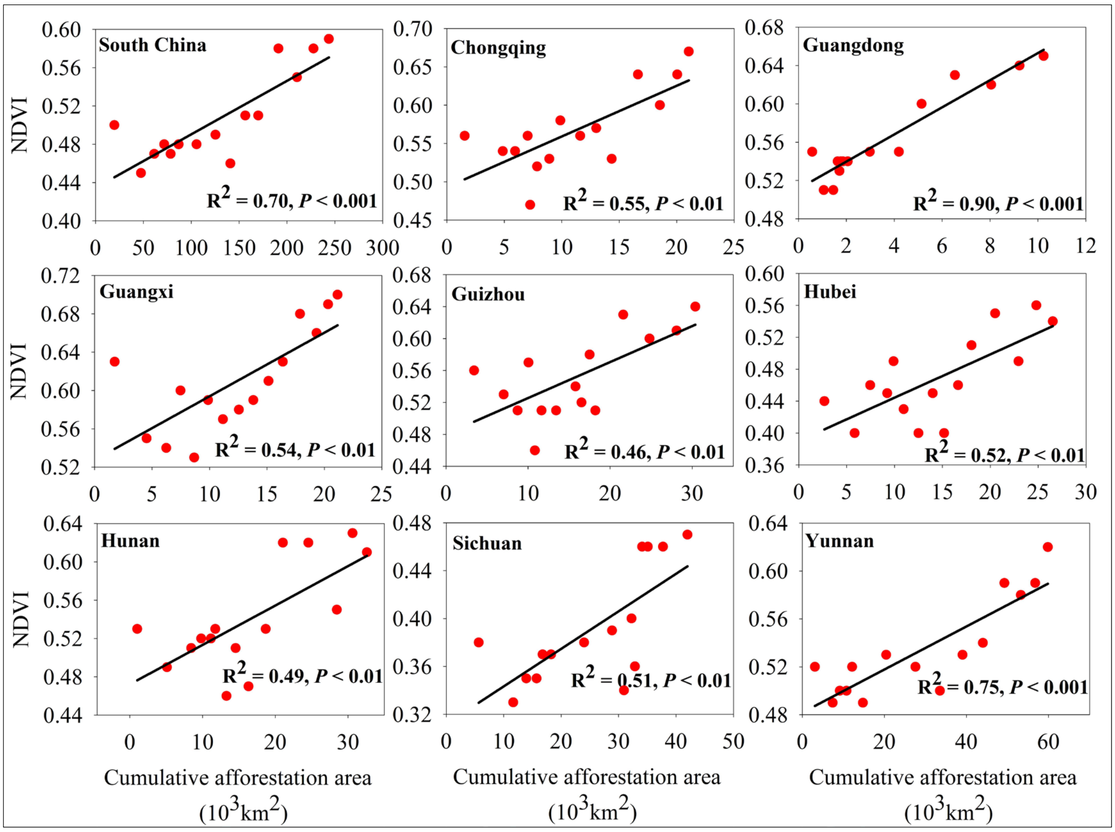

Supplementary Figure S6. Correlation between afforestation area and NDVI in South China and its eight provinces.

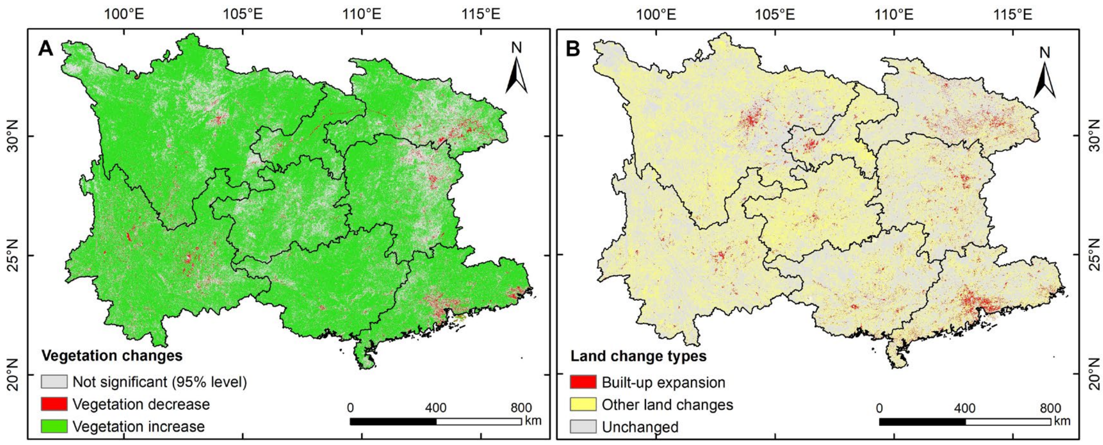

Supplementary Figure S7. Vegetation change trend and land use changes in southern China from 1987 to 2018. (a) Vegetation changes, and (b) land use changes.
